# The Effects of a Low Linoleic Acid/α-Linolenic Acid Ratio on Lipid Metabolism and Endogenous Fatty Acid Distribution in Obese Mice

**DOI:** 10.3390/ijms241512117

**Published:** 2023-07-28

**Authors:** Qiong Wang, Xingguo Wang

**Affiliations:** State Key Laboratory of Food Science and Technology, Collaborative Innovation Center of Food Safety and Quality Control in Jiangsu Province, School of Food Science and Technology, Jiangnan University, Wuxi 214122, China

**Keywords:** low linoleic acid/α-linolenic acid ratio, endogenous fatty acid, obesity, lipid metabolism

## Abstract

A reduced risk of obesity and metabolic syndrome has been observed in individuals with a low intake ratio of linoleic acid/α-linolenic acid (LA/ALA). However, the influence of a low ratio of LA/ALA intake on lipid metabolism and endogenous fatty acid distribution in obese patients remains elusive. In this investigation, 8-week-old C57BL/6J mice were randomly assigned to four groups: low-fat diet (LFD) as a control, high-fat diet (HFD), high-fat diet with a low LA/ALA ratio (HFD+H3L6), and high-fat diet with a high LA/ALA ratio (HFD+L3H6) for 16 weeks. Our results show that the HFD+H3L6 diet significantly decreased the liver index of HFD mice by 3.51%, as well as the levels of triacylglycerols (TGs) and low-density lipoprotein cholesterol (LDL-C) by 15.67% and 10.02%, respectively. Moreover, the HFD+H3L6 diet reduced the pro-inflammatory cytokines interleukin-6 (IL-6) level and aspartate aminotransferase/alanine aminotransferase (AST/ALT) ratio and elevated the level of superoxide dismutase (SOD) in the liver. The HFD+H3L6 diet also resulted in the downregulation of fatty acid synthetase (*FAS*) and sterol regulatory element binding proteins-1c (*SREBP-1c*) expression and the upregulation of peroxisome proliferator-activated receptor-α (*PPAR-α*) and acyl-CoA oxidase 1 (*ACOX1*) gene expression in the liver. The low LA/ALA ratio diet led to a notable increase in the levels of ALA and its downstream derivative docosahexaenoic acid (DHA) in the erythrocyte, liver, perienteric fat, epididymal fat, perirenal fat, spleen, brain, heart, and gastrocnemius, with a strong positive correlation. Conversely, the accumulation of LA in abdominal fat was more prominent, and a high LA/ALA ratio diet exacerbated the deposition effect of LA. In conclusion, the low LA/ALA ratio not only regulated endogenous fatty acid levels but also upregulated *PPAR-α* and *ACOX1* and downregulated *SREBP-1c* and *FAS* gene expression levels, thus maintaining lipid homeostasis. Optimizing dietary fat intake is important in studying lipid nutrition. These research findings emphasize the significance of understanding and optimizing dietary fat intake.

## 1. Introduction

With rapid global positive development, people’s diet structure and lifestyle have changed dramatically. Increased diets high in sugar and fat and reduced exercise have led to increasing rates of obesity and overweight [1]. Different types and amounts of dietary fatty acids (FAs) have different effects on the health of humans and animals. Clinical studies have shown that the type of dietary fat (or composition of FAs) impacts human health more than total dietary fat [2].

Fatty acids can regulate complex intracellular signaling systems and thus regulate cellular metabolism [3]. The replacement of saturated fatty acids (SFAs) with unsaturated fatty acids has been found to have a significant impact on reducing obesity [4]. However, the types of unsaturated fatty acids used to replace SFAs need to be further investigated. The intake of different dietary FAs can have an impact on the FA composition of an organism. There is growing evidence that the amount and type of FA in the diet affects the development of steatosis. Moreover, the type of FA accumulation in the liver may be a determinant of the progression of liver disease [5]. Excessive intake of different dietary fatty acids significantly affects the fatty acid composition of tissues. Among them, hepatic fatty acid composition closely mirrors the composition of ingested fatty acids, demonstrating congruence between dietary and liver lipid profiles. One study reported the effect of equal calories and lipids on rat tissue fatty acids. The intake of diets high in linoleic acid (LA) was found to result in a significant increase in the concentration of LA in liver phospholipids, while no significant changes were observed in the concentration of LA in the kidney [6].

Alpha-linolenic acid (ALA) and LA are essential fatty acids because they cannot be synthesized in mammals and must be consumed through the diet [7,8]. ALA and LA are precursors of the n-3 and n-3 series of polyunsaturated fatty acids (PUFAs), respectively. They are physiologically essential components of highly unsaturated fatty acids (HUFAs) and are closely related to arachidonic acid (AA) and docosahexaenoic acid (DHA) [9]. LA competes endogenously with ALA for conversion to the derivatives eicosapentaenoic acid (EPA), docosapentaenoic acid (DPA), and DHA, and excess LA also inhibits the entry of DHA and EPA into tissues. Thus, the intake of dietary FAs with different ratios of LA/ALA causes changes not only in the corresponding fatty acid levels in the body but also in their downstream products. A study found that lowering LA levels resulted in higher levels of EPA and DHA in plasma phospholipids, even if dietary ALA was kept constant [10]. A similar study came to the same conclusion and further found that AA levels in plasma phospholipids and triacylglycerols were significantly reduced if LA intake was reduced while ALA intake was increased [11]. In a systematic review, it was found that lowering LA intake and/or increasing ALA intake while decreasing LA intake to below 2.5% of total energy was effective in increasing the utilization of long-chain n-3 PUFA in vivo and also increased DHA in vivo [12]. Epidemiological studies clearly showed that an unbalanced n-6/n-3 PUFA ratio in favor of n-6 PUFA is highly pro-thrombotic and pro-inflammatory, which contributes to the prevalence of obesity, cardiovascular disease, and metabolic syndrome [13,14,15]. A prospective study clearly indicated an increase in the risk of obesity as the level of n-6 PUFA and the n-6/n-3 PUFA ratio increased in red blood cell membrane phospholipids [16]. Recent studies in humans showed that the high-ratio n-6/n-3 PUFA plays an important role in increasing the development of obesity via AA eicosanoid metabolites and hyperactivity of the cannabinoid system [17]. The conversion of n-6 and n-3 fatty acid families share the same biochemical pathway, involving a series of desaturation and elongation reaction processes [18]. Thus, there is competition between ALA and LA for metabolic enzymes, with excessive depletion of one significantly reducing the production of the other [10,19]. Balancing the ratio of LA/ALA is of great importance for obesity prevention and human health.

## 2. Results

### 2.1. A Low LA/ALA Ratio Diet Improves Metabolic Changes

Energy density refers to the energy provided per unit of food. The content of fat and carbohydrates in high-fat and low-fat diets differed significantly, and it was calculated that the food energy density of a high-fat diet was significantly higher than of a low-fat diet (*p* < 0.05). The LFD-fed group exhibited significantly greater food intake compared to the three HFD groups in Table 1 (*p* < 0.05). Because the high-fat diet is more energy dense than the basal diet, it is more likely to make the mice feel full and have a higher glycemic index, resulting in a relatively lower food intake. There was no significant difference in the average daily energy intake of the three high-fat groups (*p* > 0.05), which also indicated that the high-fat diets made with different LA/ALA ratios did not have a significant effect on the appetite of the mice.

**Figure 1:**
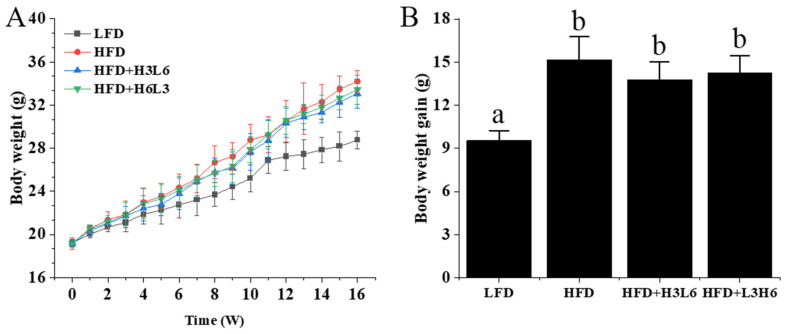
The effect of a low LA/ALA ratio on body weight and weight gain. (**A**) Body weight, (**B**) body weight gain. Data are means ± standard error (n = 10). Different letters indicate significant differences (*p* < 0.05) between each group, and the same letters indicate that there is no significant difference (*p* > 0.05) between each group.

Before starting group feeding, the initial body weights of the mice in each group were similar and not significantly different (*p* > 0.05). As the feeding time increased, the body weight of the mice in each group increased to different degrees. After 16 weeks, the mice fed a high-fat diet (HFD) experienced a 19% increase in body weight compared to those fed a low-fat diet (LFD) (*p* < 0.05), surpassing the weight gain observed in other HFD groups (as shown in Figure 1). Compared to the HFD group, the final body weight of the HFD+H3L6 and HFD+L3H6 groups decreased by 7.80% and 2.3%, respectively. The HFD+H3L6 group gained slightly less body weight than mice in the HFD+L3H6 group, but there was no significant difference (*p* > 0.05). We observed a similar trend in the amount of body weight gain (*p* > 0.05).

The organ indices of liver and fat were also important indicators of response to obesity. The results of the organ indices of mice in each group are shown in Table 2. From the results, the liver organ index, epididymis, and perirenal fat were significantly higher in the HFD group than the LFD group. Compared with the HFD group, the liver index was significantly lower in the HFD+H3L6 group (3.5070 ± 0.3271%), while the renal organ index, renal fat index, and epididymal fat index were not significantly different between the groups. As the largest metabolic organ, the low ratio LA/ALA reduced lipid accumulation in the liver, suggesting that it affects lipid metabolism in the liver and promotes lipolysis or inhibits its production.

**Figure 2:**
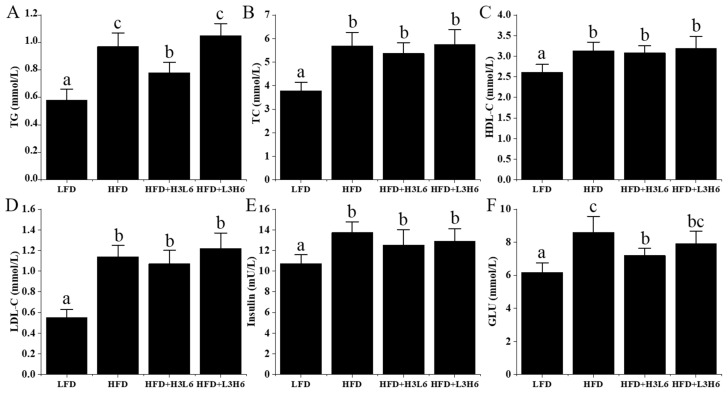
The effect of a low LA/ALA ratio on plasma lipids, insulin, and glucose. (**A**) TG; (**B**) TC; (**C**) LDL-C; (**D**) HDL-C; (**E**) insulin; (**F**) GLU. Different letters indicate significant differences (*p* < 0.05) between each group, and the same letters indicate that there is no significant difference (*p* > 0.05) between each group.

According to the data presented in Figure 2A–F, the HFD-fed mice exhibited significantly higher levels of plasma TG, TC, HDL, LDL, GLU, and insulin compared to the LFD group (*p* < 0.05). Additionally, the HFD+H3L6 group showed lower levels of plasma TG and GLU compared to the HFD-fed mice (*p* < 0.05), while TC, LDL, and insulin levels were slightly lower but not statistically significant (*p* > 0.05). Compared to the HFD+L3H6 group, the HFD+H3L6 group had significantly lower TG levels (*p* < 0.05) and slightly lower GLU levels, but they were not significant (*p* > 0.05).

### 2.2. The Effect of a Low LA/ALA Ratio Diet on the Liver

**Figure 3:**
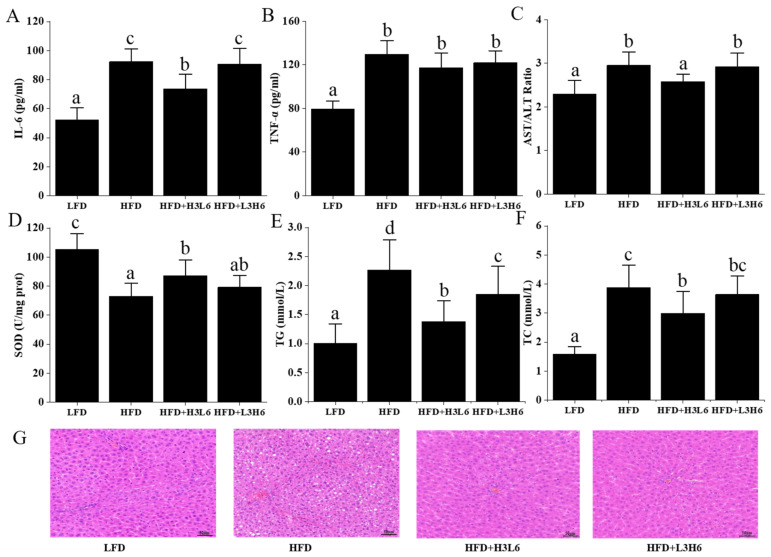
The effect of a low LA/ALA ratio on the liver. (**A**) IL-6; (**B**) TNF-α; (**C**) AST/ALT ratio; (**D**) SOD; (**E**) TG; (**F**) TC; (**G**) liver morphology (H&E). Different letters indicate significant differences (*p* < 0.05) between each group, and the same letters indicate that there is no significant difference (*p* > 0.05) between each group.

The low level of systemic inflammation that accompanies obesity, especially the inflammatory response in adipose tissue, is thought to be a major cause of insulin resistance and the development of type 2 diabetes [20,21]. The levels of inflammatory factors in mice are shown in Figure 3. Compared to the LFD group, plasma interleukin-6 (IL-6) and tumor necrosis factor-α (TNF-α) concentrations were significantly higher in the HFD group on a high-fat diet (*p* < 0.05), indicating that a high-fat diet has a pro-inflammatory effect. In the plasma of mice in the HFD+H3L6 group, IL-6 levels decreased significantly (*p* < 0.05) and TNF-α decreased slightly but not significantly (*p* < 0.05). Aspartate aminotransferase/alanine aminotransferase (AST/ALT) ratio levels can reflect the inflammatory response in the liver and are often used as indicators of liver injury in clinical practice. The HFD+H3L6 group mice had significantly lower hepatic AST/ALT ratio levels (*p* < 0.05) compared to the high-fat group. The HFD+H3L6 group of mice showed a significant decrease in liver superoxide dismutase (SOD) levels (*p* < 0.05) compared to the high-fat group. SOD is a superoxide dismutase, and enhancing its activity reduces the level of hepatic lipid peroxidation caused by obesity [22]. The results showed that both the HFD group, HFD+L3H6, and HFD+H3L6 group showed different degrees of alteration in oxidative stress indexes compared to the LFD group, indicating that a high-fat diet promotes oxidative stress in the body. Liver SOD levels in mice in the HFD+H3L6 group were significantly lower (*p* < 0.05) compared to the high-fat group. HFD+H3L6, on the other hand, exhibited significant antioxidant properties that could attenuate the liver damage caused by the high-fat diet. The results of H&E staining of liver tissues in each group of mice are shown in Figure 3G. The structure of liver lobules in the LFD group was clear. The hepatocyte cords were neatly arranged, the cells were intact, the nuclei were blue, and the cytoplasm was stained red. Compared with the LFD group, a large accumulation of white lipid droplets could be clearly seen in the HFD group, indicating that the high amounts of fat caused the formation of fat accumulation in the liver tissue of mice, which is consistent with the above-mentioned results of dyslipidemia. The HFD+H3L6 and HFD+L3H6 groups of mice had a lower number of hepatic fat vacuoles compared to the HFD group, with the HFD+H3L6 group showing a more pronounced effect than the HFD+L3H6 group.

### 2.3. The Effect of a Low LA/ALA Ratio on Lipid Metabolism Gene Expression

**Figure 4:**
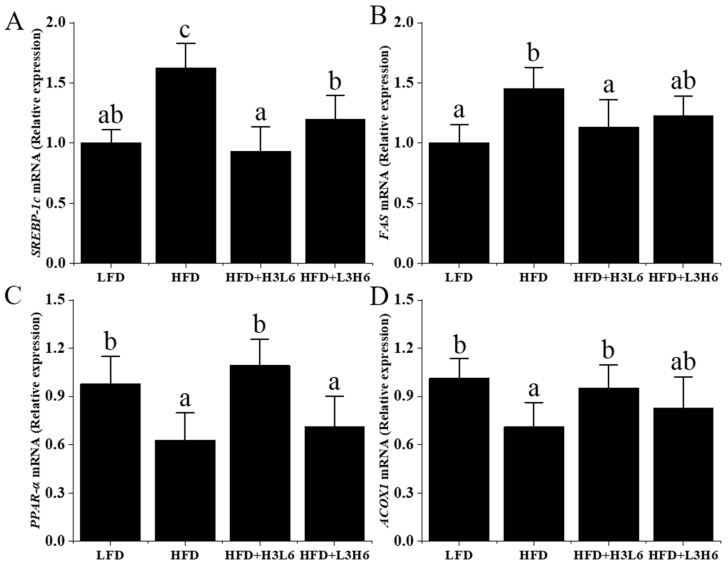
The effect of a low LA/ALA ratio on liver oxidation index and gene expression. (**A**) *SREBP-1c*; (**B**) *FAS*; (**C**) *PPAR-α*; (**D**) *ACOX1*. Different letters indicate significant differences (*p* < 0.05) between each group, and the same letters indicate that there is no significant difference (*p* > 0.05) between each group.

In order to understand how a low LA/ALA ratio affects lipid metabolism, researchers examined gene expression. The results showed that compared to the HF group (Figure 4), the other groups exhibited a significant downregulation (*p* < 0.05) in the expression of fatty acid synthase (*FAS*) and *SREBP-1c* genes. Notably, the HFD+H3L6 group demonstrated the most pronounced effect. The low LA/ALA ratio also significantly elevated acyl-CoA oxidase 1 (*ACOX1*) in lipid synthesis (*p* < 0.05). These results suggest that the presence of a low ratio of LA/ALA inhibited the downregulation of *PPAR-α* mRNA levels by a high-fat diet under high-fat diet conditions. Through the data analysis, it was observed that *ACOX1*, a downstream target involved in fatty acid oxidation, exhibited a similar pattern.

### 2.4. Tissue Fatty Acids

The liver is the most important site for the synthesis of polyunsaturated fatty acids. In addition to meeting its own needs, the liver supplies other organs through the body’s circulatory system. There is a dynamic balance between fatty acids entering the liver, fatty acids synthesized in the liver, and fatty acids processed by the liver [23], and excessive intake of a particular fatty acid can adversely affect this balance. The ratios of the major fatty acid composition in the liver are shown in Table 3. The major fatty acid compositions in the liver include palm acid, stearic acid, oleic acid, linoleic acid, dihomo-gamma-linolenic acid, arachidonic acid, and docosahexaenoic acid. The HFD group had the highest amount of OA (23.15%), the HFD+H3L6 group had the highest amount of ALA (8.55%), and the HFD+L3H6 group had the highest LA content (24.42%), and the trend remained consistent with the fatty acid content added in different high-fat dietary groups. Compared to the LFD and HFD groups, the HFD+H3L6 and HFD+L3H6 groups had lower levels of SFA and MUFA and higher levels of PUFA, regardless of the LA/ALA ratio. Regarding the conversion of polyunsaturated fatty acids, the AA content in the HFD+L3H6 group was significantly higher than in the other two high-fat diets, with an improvement rate of 102.05%. The levels of DPA and DHA in the HFD+H3L6 group were significantly elevated compared to the other two high-fat diets, with improvement rates of 45.45% and 197.80%, respectively. In terms of the improvement rate, the low LA/ALA diet ratio could greatly increase the n-3 LCPUFA content in liver tissues.

Adipose tissue stores energy and reflects the dietary fatty acid intake [24]. Table 4 presents the primary fatty acid composition in adipose tissue. The fatty acid profile and quantity in adipose tissue surrounding the intestines, epididymis, and kidneys were similar, primarily comprising palmitic acid (PA), stearic acid (SA), oleic acid (OA), and LA. Compared with the LFD group, the saturated fatty acid (SFA) and monounsaturated fatty acid (MUFA) contents of perienteric, epididymal, and perirenal fat in the HFD group were significantly higher, and the PUFA content was significantly lower. The highest ALA content was found in the HFD+H3L6 group, with 17.75%, 18.13%, and 17.16% in perienteric, epididymal, and perirenal fat, respectively. The highest LA content was found in the HFD+L3H6 group, with 38.56%, 37.98%, and 38.51% in perienteric, epididymal, and perirenal fat, respectively. The trend was generally consistent with the fatty acid content of the high-fat dietary addition. Compared to the HFD group, the HFD+H3L6 group had lower levels of PA, palmitoleic acid (POA), SA, OA, SFA, and MUFA and smaller n-6/n-3 ratios, while the LA, ALA, DHA, PUFA, n-6, and n-3 were significantly elevated. Compared to the HFD group, the HFD+L3H6 group had lower PA, POA, SA, OA, SFA, and MUFA content, whereas LA, dihomo-γ-linolenic acid (DGLA), PUFA, n-6 content, and the n-6/n-3 ratio were significantly elevated. Differences between the HFD+H3L6 and HFD+L3H6 groups were mainly concentrated in unsaturated fatty acids, and fatty acids with significant differences were OA, LA, ALA, γ-linolenic acid (GLA), DHA, MUFA, n-6, n-3, and the n-6/n-3 ratio. OA was added to the feeds of the HFD+H3L6 and HFD+L3H6 groups in nearly equal amounts (26%), but OA content in perienteric fat, epididymal fat, and perirenal fat was higher than dietary intake in the HFD+L3H6 group, probably because LA promoted the deposition of OA in adipose tissue.

In terms of PUFA metabolic transformation, adipose tissue was mainly absorbed and stored, with only trace amounts of PUFA involved in metabolism and transformation. The levels of n-6 in DGLA, AA, EPA, and DHA were lower in perienteric, epididymal, and perirenal fat, ranging from 0.03% to 0.6%. The n-6/n-3 ratios in perienteric fat, epididymal fat, and perirenal fat were significantly lower in the HFD+H3L6 group than the other three groups (1.27, 1.26, and 1.43) because their adipose tissue stored more ALA. The n-6/n-3 ratios in the HFD+H3L6 group (1.27, 1.26, and 1.43) were significantly lower than those in the other three groups because their adipose tissue stores more ALA. Fatty acid profiles in the brain, heart, gastrocnemius, spleen, and erythrocyte are shown in Tables S1–S5.

### 2.5. Relationship between Fatty Acids and Dietary Fatty Acids in Tissues

**Figure 5:**
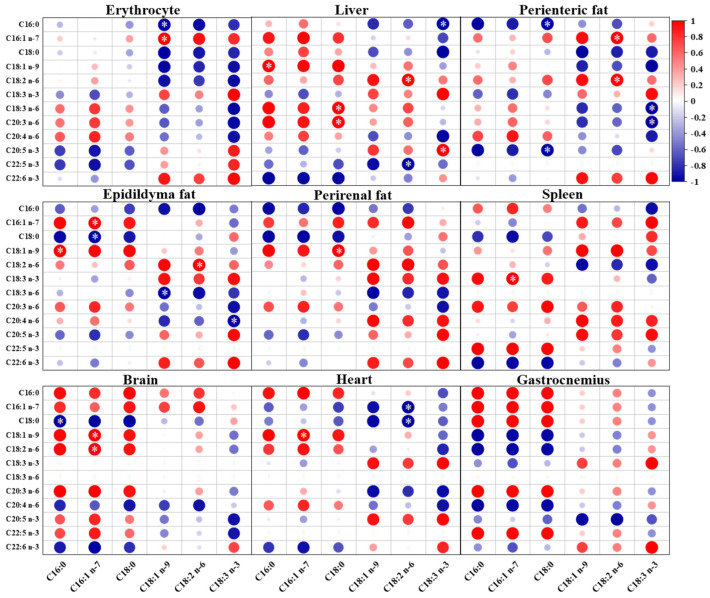
The Pearson correlation between fatty acids in the low LA/ALA ratio group and twelve fatty acids in the erythrocyte and eight organs (red denotes positive correlations and blue denotes negative correlations. The larger the circle, the higher the correlation coefficient. ∗ denotes the significant correlation (*p* < 0.05)).

**Figure 6:**
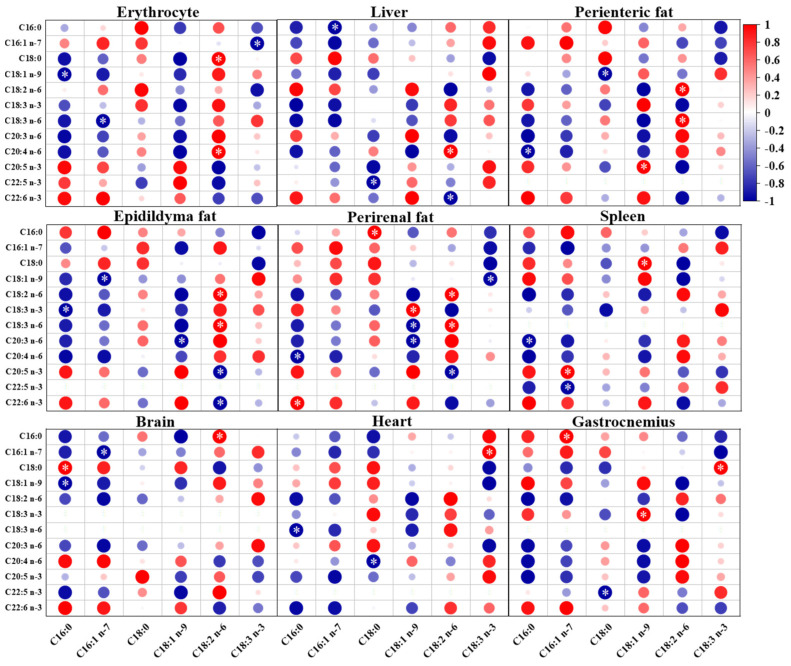
The Pearson correlation between fatty acids in the high LA/ALA ratio group and twelve fatty acids in the erythrocyte and eight organs (red denotes positive correlations and blue denotes negative correlations. The larger the circle, the higher the correlation coefficient. ∗ denotes the significant correlation (*p* < 0.05)).

As shown in Figure 5, the levels of ALA in erythrocyte and eight tissue organs of mice fed a low ratio of LA/ALA were positively correlated with the levels of ALA in dietary fatty acids. The contents of DHA and EPA in tissues and organs were also positively correlated with ALA. Except for the brain, strong negative correlations were observed between DGLA in other tissues and organs and dietary ALA. The content of LA in the liver, perienteric fat, and epididymal fat showed a strong positive correlation with dietary ALA, while the correlation in other tissues and organs was not significant. As shown in Figure 6, when feeding mice with a high LA/ALA ratio, the dietary LA was strongly and positively correlated with LA and GLA levels in perienteric fat (*p* < 0.05), epididymal fat (*p* < 0.05), and perirenal fat (*p* < 0.05), and moderately and positively correlated with LA and GLA levels in the other tissues and organs. In mice on a high LA/ALA diet, strong negative correlations were observed between dietary LA intake and AA content in the erythrocyte and liver (*p* < 0.05).

## 3. Discussion

Dietary fatty acids provide essential nutrients and meet energy requirements, but an unbalanced intake of dietary fatty acids may lead to lipid accumulation and promote obesity [25,26]. LA and ALA are essential fatty acids in the body and perform different physiological functions. However, modern diets usually have an excessive intake of LA and a relatively insufficient intake of ALA, resulting in an elevated n-6/n-3 ratio. This unbalanced fatty acid intake ratio may be associated with the development and progression of obesity [16]. Based on our findings, mice subjected to a low LA/ALA diet showed notable decreases in body weight, liver index, plasma TG, and fasting glucose levels. This indicates that the diet has a potential inhibitory effect on obesity and liver steatosis in mice. Excessive energy intake can result in fatty acid denaturation and lipid buildup in the liver. A low ratio of LA/ALA intake prevented adipocyte hypertrophy and reduced obesity [27,28]. In mice fed a high-fat diet containing a low ratio of LA/ALA, we observed a significant increase in the antioxidant capacity of the liver and a decrease in TG and TC levels in the liver. In addition, elevated AST/ALT ratio levels (2.95 ± 0.31) indicated the presence of liver damage in mice, and a low ratio of LA/ALA (2.58 ± 0.17) significantly attenuated this damage. A low LA/ALA ratio also contributes significantly to antioxidant capacity both in vitro and in vivo [29,30]. These results suggest that a low LA/ALA ratio not only reduces high-fat diet-induced obesity but also improves liver function.

High-fat diets can cause obesity and result in the buildup of lipids, oxidative stress, and chronic inflammation [31]. Mice fed a high-fat diet with a low LA/ALA ratio restored SOD activity compared with other high-fat diets. These results suggest that a low LA/ALA ratio can reduce fatty liver formation by reducing antioxidant effects, which is consistent with other reports [32,33]. In addition, supplementation with a low ratio of LA/ALA also inhibited the production of IL-6 and TNF-α. As a result of long-term high-fat dietary intervention, mice experienced fat accumulation in both subcutaneous tissue and organs, leading to an increased weight of the body and organs. Mice in the low-ratio LA/ALA-supplemented group lost 7.8% of their body weight and had significantly lower organ weights compared to the HF group. These findings indicate that the involvement of LA in lipid metabolism regulation aligns with previous research findings. (1) The high content of ALA in vegetable oils is less likely to cause obesity than LA. ALA can improve fat metabolism by regulating fatty acid oxidation and fat synthesis pathways [34]. Studies have shown that ALA intake can promote mitochondrial fatty acid oxidation and increase energy expenditure [35]. In addition, ALA can also inhibit the differentiation and proliferation of adipocytes and reduce fat synthesis. (2) ALA has anti-inflammatory effects, which can reduce the negative impact of chronic inflammation on obesity. Chronic low-grade inflammation is closely related to obesity and is involved in the development of obesity-related complications. ALA and its metabolites, such as EPA and DHA, have anti-inflammatory effects and can reduce the inflammatory response, thereby improving the metabolic disorders associated with obesity. (3) ALA may also reduce weight gain by affecting appetite and energy intake. Some studies have found that ALA can improve satiety and reduce the intake of high-calorie foods, thereby reducing energy intake.

The impact of different types of dietary fatty acids on animal health can influence lipid metabolism [36]. The liver and adipose tissue play crucial roles in maintaining energy and lipid balance [37,38,39]. In a study analyzing gene expression related to lipid metabolism and its correlation with fatty acid composition, significant alterations were observed in key genes involved in lipid homeostasis. Notably, *SREBP-1c*, *PPARα*, and *FAS* showed significant associations with varying fatty acid profiles. A low ratio of LA/ALA had a significant inhibitory effect on lipid accumulation in hepatocytes. In HepG2 hepatocyte assays, a decreased LA/ALA ratio led to increased phosphorylation of AMP-activated protein kinase (*AMPK*) and its downstream target, acetyl coenzyme A carboxylase (*ACC*) [40,41]. This phosphorylation process enhanced the activity of *AMPK*, resulting in reduced lipid accumulation in the liver. Concurrently, gene expression related to lipid metabolism provided further evidence supporting the effects of a low LA/ALA ratio. PPARα expression was suppressed in the high-ratio LA/ALA group, and *PPARα* in the low-ratio LA/ALA group showed a trend of significantly increased relative expression throughout the experiment. *PPARα* serves as a liver lipid sensor, detecting and responding to variations in fatty acids by activating the transcription of genes under its regulation [42]. In addition, it is involved in lipoprotein regulation, lipid metabolism, and glucose homeostasis [43]. Genes in the *PPARα* family, such as PPARα, can be activated by fatty acids [44]. Interestingly, we also observed the same trend for the mRNA encoding the *ACOX1* gene, a downstream target of PPARα that regulates fatty acid oxidation in vivo by peroxidases. This implies that the low ratio of LA/ALA may reduce lipid accumulation in the liver by promoting fatty acid oxidation. On the other hand, the upregulation of *SREBP-1c* and *FAS* leads to increased hepatic lipid synthesis and deposition [45,46,47]. The main role of *SREBP-1c* is to maintain lipid balance, specifically in regulating fatty acid metabolism [48]. Some studies indicate that n-3 PUFA can inhibit *SREBP-1c* by reducing mRNA transcription and promoting mRNA degradation [49], resulting in the downregulation of other genes involved in lipid metabolism, like *ACOX1* and *FAS* [50]. We found that the expression levels of *SREBP-1c* and *FAS* were downregulated after decreasing the LA/ALA ratio, indicating that low-ratio LA/ALA can inhibit lipid synthesis in hepatocytes. This result is consistent with other studies in the field of lipid metabolism and further validates the regulatory role of low-ratio LA/ALA on lipid metabolism. Based on the findings mentioned earlier, it can be concluded that maintaining a low LA/ALA ratio has the potential to regulate lipid metabolism and support liver health. This is achieved by decreasing lipid synthesis and promoting lipid oxidation. Given that fatty liver is the result of excessive lipid accumulation in hepatocytes, this finding strongly suggests that inhibition of obesity and fatty liver formation by modulating the LA/ALA ratio may be an important nutritional preventive mechanism.

In vivo, dietary fats play a significant role in adipogenesis and maintaining lipid homeostasis by influencing the composition of fatty acids [51]. However, there is a limited number of studies comparing the long-term effects of different dietary fats on lipid homeostasis. The findings of this study demonstrated a significant accumulation of ALA and LA in tissues involved in lipid metabolism in both the low and high LA/ALA ratio groups. Our findings validate and confirm earlier observations that the FA composition of lipid metabolizing tissues is significantly influenced by dietary FA composition [52,53]. The correlation analysis suggests that the liver is particularly responsive to variations in fatty acids from dietary fat. Moreover, a high LA/ALA ratio diet led to the accumulation of LA and AA, specifically in the liver. Visceral fat showed accumulations of OA, LA, DGLA, and AA, with LA being the predominant fatty acid in diets with a high LA/ALA ratio. Our findings regarding tissues enriched with LA align with a previous study, which found elevated LA concentrations in the liver but not in organs like the heart and brain. However, further investigation is needed to understand the underlying mechanisms.

Various ratios of LA/ALA in the diet led to distinct alterations in endogenous fatty acids within the liver and visceral fat. Notably, genes associated with lipid metabolism, including *PPARα* and *FAS*, displayed adaptive expression patterns in response to the introduction of exogenous fatty acids. Furthermore, a significant correlation was observed between the distribution of fatty acids and the expression of these genes. In this study, *SREBP-1c* was found to be downregulated in the tissues of both high-fat (HF) groups, with its levels inversely correlated to the LA/ALA ratio. PPARα, on the other hand, exhibited activation in the liver of both HF groups, but its levels decreased with an increasing LA/ALA ratio, ultimately resulting in an augmentation of visceral fat weight. In addition, the transcriptional profile of genes related to lipid metabolism correlated with fatty acid (LA/ALA) content. In the high ratio LA/ALA group, there was a significant negative correlation observed between the levels of OA and LA in the liver and the levels of *SREBP-1c* and *ACOX1*. In the low-ratio LA/ALA group, ALA levels in the liver were negatively correlated with *SREBP-1c,* and PPARα levels were significantly positively correlated, respectively. We found that some FA, including GLA and DGLA, were not abundant in the diet but also accumulated in the lipid metabolizing organs of high-fat-diet mice, probably due to fatty acid conversion. DGLA and AA are abundant in the tissues of the high-ratio LA/ALA group, and EPA and DHA are abundant in the tissues of the low-ratio LA/ALA group. ALA and LA are two essential fatty acids that cannot be synthesized by humans and must be obtained from the diet. LA can be endogenously converted to other n-6 PUFA, including DGLA and AA, and ALA can be endogenously converted to other n-3 PUFA, including EPA and DHA [54,55]. Although, the conversion of PUFA is inefficient in mammals.

## 4. Materials and Methods

### 4.1. Animal and Diet

A total of 40 male C57BL/6 J mice (specific pathogen-free mice; weight, 19 ± 2 g) were purchased from Viton Lever (Beijing, China) Laboratory Animal Technology Co. Animal experiments were performed under the guidance of the Animal Care and Use Committee of Jiangnan University, following the guidelines for ethical review of laboratory animal welfare. The animal experiment license number is JN. No. 20210615c0800931. The experimental mice were placed in individually ventilated cages, maintained at a constant room temperature (22–24 °C) and 60% relative humidity, and light-illuminated (12 h light/dark cycle) for a 14-day adaptation period of acclimatization feeding. All mice had free access to diets and deionized water. We gave them a standardized diet and changed the bedding and water weekly. A total of 40 mice were divided equally into 4 groups of 10 mice each. The fatty acids and main concomitants of sunflower oil, flaxseed oil, and olive oil are shown in Table 5. All edible oils and fats in the high-fat diet were replaced with a blend of sunflower oil, linseed oil, and olive oil to obtain the corresponding high LA/ALA ratio group and low LA/ALA ratio group (200 mg of tocopherol per kg of an oil blend) of the high-fat feed formulation, respectively. The detailed dietary composition of each experimental group is shown in Table 6. The first group of mice was fed a low-fat diet (LFD; 65% carbohydrate, 20% protein, and 15% fat), the second group was fed a high-fat diet (HFD; 35% carbohydrate, 20% protein and 45% fat), the third group was fed a high-fat diet with a low LA/ALA ratio (HFD+H3L6, 35% carbohydrate, 20% protein, and 45% fat), and the fourth group was fed a high-fat diet with a high LA/ALA ratio (HFD+L3H6, 35% carbohydrate, 20% protein, and 45% fat). Dietary fatty acid composition for the HFD+H3L6 group and HFD+L3H6 group are shown in Table 7.

Changes in the general signs and body weight of mice were the key indicators of their status. During the experiment, the food intake and body weight of each group of mice were weighed and measured weekly. The hair and mental status of each group of mice were observed. The mice were reared for 16 weeks and then dissected. Mice were fasted for 12 h before dissection and placed under anesthesia by intraperitoneal injection of chloral hydrate (10%, 5 mL/kg).

Blood samples were collected through extracorporeal cardiac puncture. The blood is centrifuged to obtain plasma and erythrocyte (1006.20× *g*, 15 min). The liver, perienteric fat, epididymal fat, and perirenal fat tissues, kidney, spleen, brain, heart, and gastrocnemius were dissected, weighed, frozen in liquid nitrogen, and stored in a refrigerator at −80 °C. The organ index of the liver, kidney, epididymal fat, and perirenal fat was calculated using the following formula:

Organ index (%) = organ weight (g)/mouse weight (g) × 100

Total cholesterol (TC), triacylglycerols (TG), low-density lipoprotein cholesterol (LDL-C), high-density lipoprotein cholesterol (HDL-C), blood glucose (GLU), insulin, AST, and ALT levels were measured following the instructions provided with the kit (Nanjing Institute of Biological Engineering, Nanjing, China) [56,57]. The liver was weighed and fixed in formalin, and the internal structures were examined using hematoxylin-eosin (H&E) staining.

### 4.2. Fatty Acids Extraction

Total lipids were extracted from the feeds and tissues of mice using the Folch extraction method [58]. Approximately 100 mg of the liver was added to a test tube containing 1 mL chloroform/methanol (2:1, *v*/*v*) and crushed with a homogenizer. The sample was added saline and vortexed shake for 15 min and then centrifuged at 4 °C and 10,000× g for 10 min. Using a pipette, we transferred the lower solution (fatty phase) to another test tube. The organic phase was washed 3 times with chloroform/methanol and dried under nitrogen to obtain a lipid sample. The obtained lipid was combined with 2 mL 0.5 mol/L KOH-methanol and then agitated in a water bath at 65 °C for a duration of 30 min.

Then, the extracted lipid was added to 2 mL of a 14% BF3-methanol solution and vibrated in a 70 °C water bath for 10 min. After cooling to room temperature, the samples were added to 1 mL of chromatographically pure hexane and shaken to extract fatty acid methyl esters. Following the addition of 1 mL saturated sodium chloride solution, the samples were vortexed, left to stand, and separated. The resulting supernatant was collected, filtered through a 0.45 μm organic membrane filter, and subjected to gas phase detection for analysis.

Gas chromatography was employed for fatty acid methyl ester (FAME) separation using an Agilent GC system in conjunction with a flame ionization detector (FID). The system was equipped with a silica capillary column (DB-Fast FAME, 30 m × 0.25 mm, 0.25 μm). The injector temperature was 250 °C and the hydrogen flame ion detector temperature was 260 °C. Programmed temperature rise: initial temperature of 80 °C for 0.5 min; 80 °C–165 °C, temperature rise rate of 40 °C/min, hold for 1 min; and 165 °C–230 °C, temperature rise rate of 2 °C/min, hold for 1 min. Nitrogen gas was used as the carrier gas at a flow rate of 25 mL/min and a shunt ratio of 100:1. The injection volume for analysis was set at 1.0 μL. All fatty acids were identified by comparing their retention times with those of the FAME standards. Fatty acid content was quantified using the area normalization method.

### 4.3. RNA Extraction

Liver tissue was used to extract total RNA using the Spin Column Animal Total RNA Purification Kit from Sangon Biotech, Shanghai, China. The purified RNA was then stored at −80 °C until further use. Subsequently, complementary DNA (cDNA) was synthesized from each RNA sample using the MightyScript Plus First Strand cDNA Synthesis Master Mix from Sangon Biotech, China.

Samples were analyzed using the Real-Time PCR System from Thermo. The primer sequences used for gene amplification can be found in Table 8. The PCR amplification conditions included a pre-cycle heat activation step at 95 °C for 3 min, followed by 40 cycles of denaturation at 95 °C for 5 s, and annealing and extension at 60 °C for 20 s. The mRNA levels were calculated using the 2^−ΔΔCt^ method and normalized with β-actin as the endogenous reference. The experimental primers were obtained from Shanghai Bioengineering Co., LTD. (Shanghai, China).

### 4.4. Statistical Analysis

Data analysis and statistics were performed using Microsoft Excel and PASW Statistics package version 17.0 (SPSS Inc., Chicago, IL, USA). The results are presented as mean values ± standard error mean (SEM). Differences between two or more groups were analyzed by means of Student’s *t*-test or ANOVA followed by a Bonferroni post hoc test, respectively. Correlation analysis was performed using Pearson’s algorithm and classified as a strong (r > 0.7), moderate (0.3 ≤ r ≤ 0.7), or weak (r < 0.3) correlation [59]. *p* < 0.05 was considered a statistically significant difference.

## 5. Conclusions

We investigated the effects of a low LA/ALA ratio diet on lipid metabolism and the effects of fatty acids on the FA profiles of the liver and visceral fat. The results showed that low-ratio LA/ALA inhibited weight gain and liver fat deposition in obese mice on a high-fat diet. A low ratio of LA/ALA could reduce lipids and blood glucose to some extent and ameliorate the oxidative stress caused by a high-fat diet on the liver. It suppressed liver injury by upregulating lipid metabolism gene expression and downregulating lipid synthesis gene expression. The fatty acids in the main organs of lipid metabolism (liver and abdominal fat) correlated more significantly with those in the corresponding dietary fat under different LA/ALA intervention ratios. The accumulation of LA was more significant in abdominal fat, and a diet with a low ratio of LA/ALA inhibited the deposition of LA and AA in the liver. This study provides a theoretical basis for lipid nutrition and rational intake of dietary fat.

## Figures and Tables

**Table 1:** The effects of low linoleic acid/α-linolenic acid (LA/ALA) ratio on food intake.

Group	Dietary Intake (g/d)	Food Energy Density (kcal/g)	Energy (kcal/d)
LFD	3.54 ± 0.27 ^b^	3.85 ± 0.29 ^b^	13.63 ± 0.52 ^a^
HFD	3.11 ± 0.15 ^a^	4.74 ± 0.35 ^a^	14,74 ± 0.23 ^b^
HFD+H3L6	3.01 ± 0.34 ^a^	4.74 ± 0.28 ^a^	14.27 ± 0.75 ^b^
HFD+L3H6	3.06 ± 0.31 ^a^	4.74 ± 0.33 ^a^	14.50 ± 0.65 ^b^

Data are means ± standard error (n = 10). Different letters indicate significant differences (*p* < 0.05) between each group, and the same letters indicate that there is no significant difference (*p* > 0.05) between each group.

**Table 2:** The effect of a low LA/ALA ratio on the organ indexes of mice.

Group	Organ Index (%)			
	Liver	Kidney	Perirenal Fat	Epididymal Fat
LFD	3.1964 ± 0.3875 ^a^	1.0668 ± 0.1079 ^a^	1.0153 ± 0.1295 ^a^	2.1396 ± 0.3409 ^a^
HFD	3.9544 ± 0.1915 ^c^	1.0845 ± 0.0871 ^a^	1.4586 ± 0.1289 ^b^	3.6562 ± 0.3016 ^b^
HFD+H3L6	3.5070 ± 0.3271 ^ab^	1.0363 ± 0.1029 ^a^	1.1430 ± 0.1032 ^b^	3.4719 ± 0.4722 ^b^
HFD+L3H6	3.7583 ± 0.2756 ^bc^	1.0292 ± 0.0571 ^a^	1.2776 ± 0.1347 ^b^	3.5854 ± 0.3911 ^b^

Data are means ± standard error (n = 10). Different letters indicate significant differences (*p* < 0.05) between each group, and the same letters indicate that there is no significant difference (*p* > 0.05) between each group.

**Table 3:** Fatty acid composition of the liver (%).

Fatty Acids	LFD	HFD	HFD+H3L6	HFD+L3H6
C14:0	0.58 ± 0.07	0.62 ± 0.06	0.63 ± 0.07	0.61 ± 0.05
C16:0	19.42 ± 0.72 ^b^	19.45 ± 1.83 ^b^	15.94 ± 0.64 ^a^	15.54 ± 0.96 ^a^
C16:1 n-7	0.95 ± 0.05	1.01 ± 0.88	0.83 ± 0.16	0.20 ± 0.05
C18:0	13.57 ± 0.68	13.81 ± 0.72	12.38 ± 0.29	12.92 ± 1.30
C18:1 n-9	18.77 ± 1.04 ^b^	23.15 ± 1.06 ^c^	11.67 ± 0.82 ^a^	11.03 ± 1.36 ^a^
C18:2 n-6	19.57 ± 0.72 ^a^	18.29 ± 1.21 ^a^	18.95 ± 1.45 ^a^	24.42 ± 0.75 ^b^
C18:3 n-3	0.70 ± 0.27 ^a^	0.26 ± 0.14 ^a^	8.55 ± 0.70 ^b^	0.28 ± 0.08 ^a^
C18:3 n-6	0.37 ± 0.10	0.30 ± 0.04	0.39 ± 0.46	0.43 ± 0.11
C20:0	0.43 ± 0.02	0.47 ± 0.06	0.45 ± 0.01	0.48 ± 0.04
C20:1 n-9	0.61 ± 0.01	0.66 ± 0.06	0.63 ± 0.06	0.56 ± 0.02
C20:2 n-6	0.25 ± 0.01	0.26 ± 0.01	0.25 ± 0.02	0.23 ± 0.02
C20:3 n-6	4.86 ± 0.45	4.78 ± 0.67	5.16 ± 0.84	5.12 ± 0.55
C20:4 n-6	8.54 ± 1.02 ^b^	7.81 ± 1.14 ^b^	9.23 ± 0.51 ^a^	15.77 ± 0.80 ^b^
C20:5n-3	0.67 ± 0.06 ^a^	1.67 ± 0.14 ^b^	0.73 ± 0.11 ^a^	1.91 ± 0.14 ^b^
C22:0	0.07 ± 0.01	0.08 ± 0.01	0.08 ± 0.01	0.07 ± 0.01
C22:1 n-9	0.06 ± 0.01	0.05 ± 0.01	0.05 ± 0.01	0.06 ± 0.01
C22:5 n-6	0.44 ± 0.03	0.44 ± 0.03	0.44 ± 0.02	0.48 ± 0.07
C22:5 n-3	0.75 ± 0.70 ^c^	0.55 ± 0.04 ^b^	0.80 ± 0.01 ^c^	0.19 ± 0.06 ^a^
C22:6 n-3	4.52 ± 0.24 ^b^	1.82 ± 0.10 ^a^	5.42 ± 0.17 ^c^	1.66 ± 0.10 ^a^
SFA	34.06 ± 0.33 ^b^	34.44 ± 2.49 ^b^	29.47 ± 1.00 ^a^	29.62 ± 0.52 ^a^
MUFA	20.51 ± 0.98 ^b^	25.00 ± 1.28 ^c^	13.30 ± 0.82 ^a^	11.99 ± 1.41 ^a^
PUFA	40.78 ± 1.14 ^a^	36.93 ± 2.81 ^a^	49.77 ± 1.63 ^b^	50.59 ± 1.45 ^b^
n-6	34.15 ± 1.38 ^a^	32.62 ± 3.24 ^a^	34.54 ± 2.50 ^a^	46.55 ± 2.42 ^b^
n-3	6.63 ± 0.40 ^b^	4.31 ± 0.15 ^a^	15.23 ± 0.44 ^c^	4.04 ± 0.34 ^a^
n-6/n-3	5.18 ± 0.46 ^b^	7.58 ± 0.64 ^c^	2.27 ± 0.19 ^a^	11.57 ± 0.79 ^d^

Data are means ± standard error (n = 10). Different letters indicate significant differences (*p* < 0.05) between each group, and the same letters indicate that there is no significant difference (*p* > 0.05) between each group.

**Table 4:** Fatty acid composition of adipose tissue (%).

Fatty Acids	LFD	HFD	HFD+H3L6	HFD+L3H6
Perienteric fat				
C16:0	21.25 ± 1.44 ^b^	25.52 ± 0.24 ^c^	17.62 ± 1.25 ^a^	16.74 ± 0.56 ^a^
C16:1 n-7	2.54 ± 0.43 ^b^	2.28 ± 0.60 ^b^	0.81 ± 0.23 ^a^	0.98 ± 0.10 ^a^
C18:0	2.96 ± 0.64 ^ab^	5.43 ± 0.63 ^c^	3.71 ± 0.48 ^b^	2.34 ± 0.44 ^a^
C18:1 n-9	28.77 ± 0.39 ^b^	37.97 ± 2.66 ^c^	25.03 ± 0.80 ^a^	28.65 ± 0.92 ^b^
C18:2 n-6	32.32 ± 1.63 ^c^	15.88 ± 0.45 ^a^	21.28 ± 0.37 ^b^	38.56 ± 2.01 ^d^
C18:3 n-3	1.06 ± 0.06 ^ab^	0.74 ± 0.10 ^a^	17.75 ± 0.91 ^b^	0.45 ± 0.15 ^a^
C18:3 n-6	0.20 ± 0.02 ^a^	0.18 ± 0.02 ^a^	1.12 ± 0.06 ^b^	0.21 ± 0.02 ^a^
C20:3 n-6	0.38 ± 0.02^b^	0.18 ± 0.01 ^a^	0.16 ± 0.01 ^a^	0.52 ± 0.01 ^c^
C20:4 n-6	0.15 ± 0.04	0.16 ± 0.04	0.19 ± 0.01	0.13 ± 0.02
C20:5 n-3	0.22 ± 0.02 ^c^	0.04 ± 0.01 ^a^	0.06 ± 0.01 ^a^	0.14 ± 0.02 ^b^
C22:6 n-3	0.19 ± 0.03 ^c^	0.04 ± 0.01 ^a^	0.12 ± 0.01 ^b^	0.06 ± 0.02 ^a^
SFA	24.22 ± 2.06 ^b^	30.95 ± 0.86 ^c^	21.33 ± 1.45 ^ab^	19.08 ± 1.00 ^a^
MUFA	31.31 ± 0.78 ^b^	40.25 ± 2.97 ^c^	25.84 ± 1.03 ^a^	29.63 ± 0.91 ^ab^
PUFA	34.52 ± 1.69 ^b^	17.22 ± 0.29 ^a^	40.68 ± 1.13 ^c^	40.06 ± 2.01 ^c^
n-6	33.05 ± 1.62 ^c^	16.40 ± 0.41 ^a^	22.76 ± 0.40 ^b^	39.42 ± 1.93 ^d^
n-3	1.47 ± 0.12 ^a^	0.82 ± 0.12 ^a^	17.93 ± 0.91 ^b^	0.64 ± 0.15 ^a^
n-6/n-3	22.59 ± 1.44 ^b^	20.44 ± 3.21 ^b^	1.27 ± 0.0 6 ^a^	64.69 ± 12.09 ^c^
Epididymal fat				
C16:0	20.93 ± 1.41 ^b^	25.59 ± 0.29^c^	18.00 ± 1.33 ^a^	16.51 ± 0.64 ^a^
C16:1 n-7	2.82 ± 0.17 ^b^	2.88 ± 0.52 ^b^	0.92 ± 0.25 ^a^	1.06 ± 0.13 ^a^
C18:0	2.73 ± 0.55 ^a^	4.57 ± 0.74 ^b^	3.45 ± 0.33 ^ab^	2.24 ± 0.41 ^a^
C18:1 n-9	29.07 ± 0.44 ^a^	38.45 ± 2.87 ^b^	25.38 ± 0.69 ^a^	29.13 ± 1.02 ^a^
C18:2 n-6	32.12 ± 1.68 ^c^	15.37 ± 0.44 ^a^	20.30 ± 2.07 ^b^	37.98 ± 2.20 ^d^
C18:3 n-3	1.12 ± 0.10 ^a^	0.86 ± 0.13 ^a^	18.13 ± 3.88 ^b^	0.60 ± 0.23 ^a^
C18:3 n-6	0.20 ± 0.02 ^a^	0.18 ± 0.02 ^a^	1.13 ± 0.06 ^b^	0.23 ± 0.03 ^a^
C20:3 n-6	0.40 ± 0.02 ^ab^	0.19 ± 0.01 ^a^	0.17 ± 0.03 ^a^	0.66 ± 0.02 ^b^
C20:4 n-6	0.16 ± 0.04	0.18 ± 0.03	0.19 ± 0.02	0.14 ± 0.02
C20:5 n-3	0.26 ± 0.07 ^c^	0.06 ± 0.02 ^a^	0.07 ± 0.02 ^a^	0.13 ± 0.01 ^b^
C22:6 n-3	0.22 ± 0.04 ^c^	0.06 ± 0.01 ^a^	0.12 ± 0.01 ^b^	0.08 ± 0.03 ^ab^
SFA	23.66 ± 1.95 ^b^	30.16 ± 0.87 ^c^	21.44 ± 1.49 ^ab^	18.75 ± 1.03 ^a^
MUFA	31.89 ± 0.61 ^b^	41.34 ± 3.35 ^c^	26.30 ± 0.94 ^ab^	30.20 ± 1.05 ^a^
PUFA	34.48 ± 1.69 ^a^	16.89 ± 0.25 ^b^	40.10 ± 3.90 ^a^	39.82 ± 2.05 ^a^
n-6	32.88 ± 1.66 ^c^	15.93 ± 0.41 ^a^	21.79 ± 1.99 ^b^	39.01 ± 2.04 ^d^
n-3	1.60 ± 0.20 ^a^	0.97 ± 0.16 ^a^	18.31 ± 3.89 ^b^	0.81 ± 0.27 ^a^
n-6/n-3	20.87 ± 2.78 ^b^	16.98 ± 3.37 ^b^	1.26 ± 0.32 ^a^	53.63 ± 17.23 ^c^
Perirenal fat				
C16:0	21.11 ± 1.38 ^b^	25.20 ± 0.53 ^c^	17.68 ± 1.22 ^a^	16.80 ± 0.62 ^a^
C16:1 n-7	2.85 ± 0.26 ^b^	2.59 ± 0.09 ^b^	0.84 ± 0.20 ^a^	1.00 ± 0.07 ^a^
C18:0	2.74 ± 0.54 ^ab^	4.74 ± 0.49 ^c^	3.46 ± 0.32 ^b^	2.39 ± 0.41 ^a^
C18:1 n-9	28.85 ± 0.27 ^b^	38.76 ± 2.55 ^c^	25.99 ± 0.38 ^a^	28.90 ± 0.91 ^b^
C18:2 n-6	32.32 ± 1.63 ^c^	15.70 ± 0.04 ^a^	21.84 ± 1.72 ^b^	38.51 ± 2.00 ^d^
C18:3 n-3	1.06 ± 0.06 ^a^	0.77 ± 0.05 ^a^	17.16 ± 3.93 ^b^	0.46 ± 0.13 ^a^
C18:3 n-6	0.19 ± 0.02 ^a^	0.19 ± 0.02 ^a^	1.07 ± 0.09 ^b^	0.21 ± 0.01 ^a^
C20:3 n-6	0.38 ± 0.02 ^b^	0.17 ± 0.02 ^a^	0.15 ± 0.03 ^a^	0.51 ± 0.12 ^b^
C20:4 n-6	0.15 ± 0.04	0.20 ± 0.04	0.19 ± 0.01	0.14 ± 0.02
C20:5 n-3	0.20 ± 0.06 ^b^	0.04 ± 0.02 ^a^	0.05 ± 0.02 ^a^	0.11 ± 0.03 ^a^
C22:6 n-3	0.20 ± 0.01 ^c^	0.05 ± 0.01 ^a^	0.11 ± 0.02 ^b^	0.06 ± 0.01 ^a^
SFA	23.85 ± 1.90 ^b^	29.94 ± 1.01 ^c^	21.14 ± 1.51 ^ab^	19.06 ± 0.99 ^a^
MUFA	31.71 ± 0.32 ^b^	41.35 ± 2.61 ^c^	26.83 ± 0.39 ^a^	30.36 ± 1.62 ^ab^
PUFA	34.50 ± 1.69 ^b^	17.10 ± 0.04 ^a^	40.57 ± 3.81 ^c^	40.00 ± 2.01 ^c^
n-6	33.04 ± 1.62 ^c^	16.25 ± 0.07 ^a^	23.25 ± 1.60 ^b^	39.38 ± 1.92 ^d^
n-3	1.46 ± 0.13 ^a^	0.85 ± 0.06 ^a^	17.32 ± 3.94 ^b^	0.63 ± 0.17 ^a^
n-6/n-3	22.72 ± 1.68 ^b^	19.19 ± 1.33 ^b^	1.43 ± 0.38 ^a^	66.30 ± 13.86 ^c^

Data are means ± standard error (n = 10). Different letters indicate significant differences (*p* < 0.05) between each group, and the same letters indicate that there is no significant difference (*p* > 0.05) between each group.

**Table 5:** The principal fatty acids and concomitants of three oils.

	Flaxseed Oil	Sunflower Oil	Olive Oil
C16:0 (%)	5.83	6.75	9.83
C16:1 (%)	0.66	1.00	0.80
C18:0 (%)	4.23	3.95	3.30
C18:1 n-9 (%)	18.77	25.29	77.55
C18:2 n-6 (%)	20.34	60.86	5.51
C18:3 n-3 (%)	47.00	0.10	0.64
C20:0 (%)	1.27	0.05	0.41
SFA (%)	11.33	10.75	13.54
MUFA (%)	19.43	26.29	78.35
PUFA (%)	67.34	60.96	6.15
Polyphenol (mg GAE/kg)	62.46	83.51	193.25
Tocopherol (mg/kg)	352.59	627.59	189.89

**Table 6:** Diet composition.

	LFD		HFD		HFD+H3L6		HFD+L3H6	
	g/kg	kcal/kg	g/kg	kcal/kg	g/kg	kcal/kg	g/kg	kcal/kg
Casein	189.6	758.4	233.06	932.24	233.06	932.24	233.06	932.24
L-cystine	2.8	11.2	3.5	13.98	3.5	13.98	3.5	13.98
Corn starch	298.6	1194.4	84.83	339.33	84.83	339.33	84.83	339.33
Maltodextrin	33.2	132.8	116.53	466.12	116.53	466.12	116.53	466.12
Sucrose	331.7	1326.8	201.36	805.45	201.36	805.45	201.36	805.45
Cellulose	47.4	0	58.26	0	58.26	0	58.26	0
Corn oil	42.52	382.68	0	0	0	0	0	0
Lard	0	0	235.79	2123.73	0	0	0	0
Sunflower oil	0	0	0	0	64.2	577.8	221.78	1996.02
Flaxseed oil	0	0	0	0	151.75	1365.75	14.01	126.09
Olive oil	0	0	0	0	19.84	178.56	0	0
Mineral premix	9.5	8.47	11.65	10.39	11.65	10.39	11.65	10.39
Potassium hydrogen phosphate	12.3	0	15.15	0	15.15	0	15.15	0
Calcium carbonate	5	0	6.41	0	6.41	0	6.41	0
Potassium citrate	15.6	0	19.23	0	19.23	0	19.23	0
Vitamin premix	9.5	37.04	11.65	45.42	11.65	45.42	11.65	45.42
Choline bitartrate	2.2		2.5	0	2.5	0	2.5	0
TBHQ	0.02	0	0.02	0	0.02	0	0.02	0
Blue dyes	0.06	0	0.06	0	0.06	0	0.06	0
Total	1000	3851.79	1000	4736.66	1000	4735.04	1000	4735.04
Energy supply ratio (%)								
Protein	20.3	20%	24	20%	24	20%	24	20%
Carbohydrates	64.82	65%	41	35%	41	35%	41	35%
Fat	7	15%	24	45%	24	45%	24	45%

**Table 7:** Dietary fatty acid composition.

	HFD+H3L6	HFD+L3H6
C16:0	6.48	6.77
C18:0	4.03	4.01
C18:1 n-9	25.25	25.18
C18:2 n-6	29.84	59.11
C18:3 n-3	30.04	2.93
LA/ALA Ratio	1:1	20:1
Polyphenol (mg GAE/kg)	74.49	77.61
Tocopherol (mg/kg)	590.69	576.37

**Table 8:** The primer sequence used for quantitative RT-PCR.

Genes	Forward Primer (5′→3′)	Reverse Prime (5′→3′)
*FAS*	CTACGCAGGCCTTCTGAGTT	TTGCATACTCACACGACTGG
*PPARα*	CTAATCCTGACACCGGACGC	GGAGACAGGTTGTCATCGCT
*SREBP1c*	AAGGACCCTTGCGATCTGTG	GTGGTATCGGTGAGTGGCAA
*ACOX1*	TAACGCTGGCTTCGAGTGAG	CGTCCGGTGTCAGGATTAGG

## Data Availability

The data presented in this study are available on request from the corresponding author.

## Supplementary Materials

The following supporting information can be downloaded at: https://www.mdpi.com/article/10.3390/ijms241512117/s1.
