# Supplementary material for: The Effects of a Low Linoleic Acid/α-Linolenic Acid Ratio on Lipid Metabolism and Endogenous Fatty Acid Distribution in Obese Mice
Source: Int J Mol Sci. 2023 Jul 28;24(15):12117. doi: 10.3390/ijms241512117 (PMC10419107; doi:10.3390/ijms241512117)
Supplement: Supplementary file 1 [file ijms-24-12117-s001.zip › ijms-2504159-supplementary.pdf]

Table S1. Fatty acid composition of brain (%).

Fatty acids	LFD	HFD	HFD+H3L6	HFD+L3H6
C16:0	20.63±1.76	22.50±1.45	19.94±1.29	20.50±1.51
C16:1 n-7	0.43±0.02	0.38±0.03	0.38±0.05	0.34±0.04
C18:0	20.52±1.78	18.69±0.60	19.42±0.46	19.84±0.92
C18:1 n-9	19.64±0.72	19.41±0.37	18.75±0.32	18.60±0.81
C18:2 n-6	0.91±0.14	0.91±0.05	0.72±0.25	1.10±0.41
C18:3 n-3				
C18:3 n-6				
C20:3 n-6	0.75±0.27	0.55±0.20	0.85±0.33	0.55±0.17
C20:4n-6	859±1.94	9.48±2.18	8.09±2.01	9.05±1.58
C20:5n-3		0.11±0.02	0.12±0.02	0.10±0.02
C22:5n-3		0.14±0.01	0.17±0.02	0.15±0.02
C22:6n-3	10.30±0.92	10.77±1.11	10.89±0.43	10.18±0.67
SFA	41.14±1.85	41.19±1.70	39.35±0.90	40.34±0.71
MUFA	20.07±0.73	19.79±0.39	19.13±0.37	18.95±0.84
PUFA	20.62±2.40	21.91±2.36	21.84±0.59	21.46±0.82
n-6	10.25±1.54	10.94±2.03	9.66±0.29	11.04±0.63
n-3	10.30±0.92	10.98±1.09	11.18±0.41	10.43±0.64
n-6/n-3	0.99±0.06	1.01±0.20	0.79±0.03	1.06±0.06

* Data are means ± standard error (n = 10). Different letters indicate significant differences ($P<0.05$) between each group, and the same letters indicate that there is no significant difference ($P>0.05$) between each group.

Table S2. Fatty acid composition of heart (%).

Fatty acids	LFD	HFD	HFD+H3L6	HFD+L3H6
C16:0	13.77±0.92	11.60±1.44	11.17±1.92	10.57±0.91
C16:1 n-7	0.17±0.01[b]	0.07±0.01[a]	0.16±0.04[b]	0.13±0.01[b]
C18:0	19.62±2.53	19.49±1.12	18.66±1.29	18.88±1.31
C18:1 n-9	6.23±1.42	6.15±0.97	6.26±1.08	6.28±0.98
C18:2 n-6	17.99±1.12	17.65±1.46	17.57±0.79	19.22±1.59
C18:3 n-3	0.43±0.08[b]	0.03±0.01[a]	1.44±0.10[c]	0.04±0.01[a]
C18:3 n-6			0.17±0.05	
C20:3 n-6	8.80±1.03	9.08±1.04	9.81±1.21	10.62±0.64
C20:4n-6	9.53±1.64[a]	13.94±0.94[b]	9.02±0.91[a]	10.83±0.87[b]
C20:5n-3	1.37±0.33[ab]	1.80±0.65[ab]	1.07±0.17[a]	2.24±0.23[b]
C22:5n-3				
C22:6n-3	11.94±0.96[bc]	10.07±0.91[ab]	12.55±0.88[c]	8.93±1.09[a]
SFA	33.39±3.27	31.09±0.75	29.83±0.64	29.45±1.49
MUFA	6.40±1.41	6.22±0.97	6.43±1.24	6.42±0.98
PUFA	50.27±2.80	52.58±1.69	53.00±0.85	54.00±0.34
n-6	36.53±2.48[a]	40.67±1.86[bc]	37.95±0.91[ab]	42.79±1.09[c]
n-3	13.74±1.11[bc]	11.90±0.59[ab]	15.06±1.05[c]	11.21±0.87[a]
n-6/n-3	2.68±0.29[a]	3.43±0.28[b]	2.53±0.19[a]	3.85±0.41[b]

* Data are means ± standard error (n = 10). Different letters indicate significant differences (*P<0.05*) between each group, and the same letters indicate that there is no significant difference (*P>0.05*) between each group.

Table S3. Fatty acid composition of gastrocnemius (%).

Fatty acids	LFD	HFD	HFD+H3L6	HFD+L3H6
C16:0	25.84±3.40	21.24±3.73	20.69±2.82	19.14±2.74
C16:1 n-7	0.77±0.07[c]	0.52±0.02[b]	0.32±0.04[a]	0.42±0.04[ab]
C18:0	7.61±1.44[a]	10.91±0.29[b]	8.26±1.11[a]	8.59±0.58[a]
C18:1 n-9	18.74±1.42[c]	16.97±2.21[bc]	13.17±1.89[ab]	11.36±0.91[a]
C18:2 n-6	17.40±0.68[a]	19.52±1.63[a]	20.39±4.16[a]	27.19±1.67[b]
C18:3 n-3	0.48±0.10[a]	0.42±0.09[a]	6.24±1.72[b]	0.63±0.07[a]
C18:3 n-6				
C20:3 n-6	7.85±1.51[b]	3.60±0.97[a]	4.63±1.34[a]	5.35±1.10[ab]
C20:4n-6	3.91±0.79[a]	7.07±0.76[b]	2.17±0.74[a]	7.82±0.72[b]
C20:5n-3	0.40 ±0.27	0.39±0.26	0.15±0.10	0.70±0.48
C22:5n-3	0.37±0.03[a]	1.08±0.16[b]	0.37±0.13[a]	0.91±0.06[b]
C22:6n-3	11.70±1.39[c]	7.43±0.59[bc]	9.91±1.53[bc]	6.47±1.06[a]
SFA	33.45±1.96	32.15±3.44	28.95±1.97	27.73±3.32
MUFA	19.51±1.48[a]	17.49±2.19[a]	13.49±1.89[b]	11.78±0.87[b]
PUFA	42.10±3.14[ab]	39.51±1.27[a]	43.86±4.66[ab]	49.06±2.70[b]
n-6	29.15±1.61[a]	30.19±1.84[a]	27.19±5.85[a]	40.36±2.06[b]
n-3	12.95±1.53[b]	9.32±0.57[a]	16.67±0.54[c]	8.70±1.41[a]
n-6/n-3	2.27±0.14[a]	3.26±0.40[b]	1.62±0.32[a]	4.73±0.54[c]

* Data are means ± standard error (n = 10). Different letters indicate significant differences ($P<0.05$) between each group, and the same letters indicate that there is no significant difference ($P>0.05$) between each group.

Table S4. Fatty acid composition of spleen (%).

Fatty acids	LFD	HFD	HFD+H3L6	HFD+L3H6
C16:0	27.45±1.00^{c}	23.66±0.92^{b}	20.22±2.60^{a}	19.06±1.61^{a}
C16:1 n-7	0.56±0.07^{a}	0.55±0.09^{a}	2.71±0.66^{b}	0.55±0.05^{a}
C18:0	13.25±0.6	13.71±0.74	12.77±1.90	13.36±0.93
C18:1 n-9	18.67±0.91	18.28±0.90	17.58±0.74	17.54±1.00
C18:2 n-6	10.49±0.83^{b}	8.33±0.49^{a}	10.22±0.84^{b}	13.26±0.710^{c}
C18:3 n-3	0.57±0.32^{a}	0.63±0.08^{a}	3.95±0.54^{b}	0.61±0.10^{a}
C18:3 n-6				
C20:3 n-6	6.36±0.61^{a}	9.42±0.87^{b}	6.22±0.56^{a}	9.29±0.49^{b}
C20:4n-6	8.95±1.15^{a}	11.58±0.94^{b}	8.44±0.60^{a}	11.42±1.18^{b}
C20:5n-3	1.24±0.80	0.96±0.24	0.58±0.07	1.25±0.24
C22:5n-3	1.02±0.16^{a}	0.95±0.11^{a}	1.97±0.50^{b}	0.97±0.11^{a}
C22:6n-3	1.93±0.43^{a}	1.21±0.14^{a}	3.89±0.14^{b}	1.84±0.61^{a}
SFA	40.69±0.96^{c}	37.37±0.39^{b}	32.98±4.44^{a}	32.42±0.75^{a}
MUFA	19.23±0.97^{ab}	18.84±0.81^{ab}	20.29±0.60^{b}	18.08±1.04^{a}
PUFA	30.55±0.86^{a}	33.10±0.83^{b}	35.26±1.54^{b}	38.62±0.93^{c}
n-6	25.80±0.83^{a}	33.20±0.72^{b}	24.87±1.03^{a}	33.96±0.90^{c}
n-3	4.75±1.44^{a}	3.76±0.38^{a}	10.39±0.81^{b}	4.66±0.48^{a}
n-6/n-3	6.07±2.17^{b}	7.86±0.68^{b}	2.41±0.19^{a}	7.38±0.88^{b}

* Data are means ± standard error (n = 10). Different letters indicate significant differences ($P<0.05$) between each group, and the same letters indicate that there is no significant difference ($P>0.05$) between each group.

Table S5. Fatty acid composition of erythrocyte (%).

Fatty acids	LFD	HFD	HFD+H3L6	HFD+L3H6
C16:0	30.76±2.40^{c}	28.85±1.38^{ab}	25.81±0.83^{a}	27.25±1.15^{ab}
C16:1 n-7	0.56±0.06	0.42±0.12	0.47±0.07	0.44±0.06
C18:0	15.12±1.58^{b}	9.94±1.19^{a}	10.72±1.38^{a}	12.21±1.31^{ab}
C18:1 n-9	7.97±1.03^{a}	14.49±1.52^{b}	13.89±1.85^{b}	12.83±1.55^{b}
C18:2 n-6	9.65±1.33	9.20±0.30	8.57±0.28	9.89±0.39
C18:3 n-3	0.03± 0.01^{a}	0.17±0.03^{a}	0.45±0.12^{b}	0.16±0.03^{a}
C18:3 n-6	2.08±0.41^{b}	0.77±0.31^{a}	1.73±0.33^{ab}	2.56±0.83^{b}
C20:3 n-6	3.12±0.12^{a}	3.49±0.23^{ab}	3.23±0.14^{ab}	3.63±0.28^{b}
C20:4 n-6	5.82±1.22^{a}	9.13±1.12^{b}	6.72±2.09^{a}	11.05±1.39^{b}
C20:5 n-3	0.13±0.01^{a}	0.13±0.01^{a}	0.31±0.10^{ab}	0.35±0.07^{b}
C22:5 n-3	0.47±0.12	0.54±0.07	0.64±0.12	0.63±0.11
C22:6 n-3	1.85±0.41^{a}	1.73±0.57^{a}	5.05±0.85^{b}	2.13±0.57^{a}
SFA	47.67±1.05^{b}	40.37±0.26^{a}	39.16±1.72^{a}	41.07±2.22^{a}
MUFA	9.40±1.03^{a}	15.81±1.47^{b}	15.22±1.73^{ab}	14.20±1.42^{b}
PUFA	23.01±1.74^{a}	25.57±1.64^{b}	26.98±1.34^{ab}	31.46±2.32^{b}
n-6	20.38±1.29^{ab}	22.66±1.44^{b}	20.36±0.60^{a}	28.04±1.71^{c}
n-3	2.63±0.53^{a}	2.88±0.23^{a}	6.63±2.05^{b}	3.42±0.61^{a}
n-6/n-3	8.07±1.18^{b}	7.91±0.36^{b}	3.21±2.35^{a}	8.47±1.19^{b}

* Data are means ± standard error (n = 10). Different letters indicate significant differences ($P<0.05$) between each group, and the same letters indicate that there is no significant difference ($P>0.05$) between each group.
